# Derivation and validation of sex-specific continuous metabolic syndrome scores for the Mexican adult population

**DOI:** 10.1038/s41598-022-10963-w

**Published:** 2022-06-10

**Authors:** Eduardo Pérez-Castro, Flaviano Godínez-Jaimes, Martín Uriel Vázquez-Medina, María Esther Ocharan-Hernández, Cruz Vargas-De-León

**Affiliations:** 1 Maestría en Matemáticas Aplicadas, Facultad de Matemáticas, Universidad Autónoma de Guerrero, Chilpancingo, Guerrero Mexico; 2 Sección de Estudios de Posgrado e Investigación, Escuela Superior de Medicina, Instituto Politécnico Nacional, Mexico City, Mexico; 3 Departamento de Investigación, Hospital Central Militar, Secretaria de la Defensa Nacional, Mexico City, Mexico; 4 Divisón de Investigación, Hospital Juárez de México, Mexico City, Mexico

**Keywords:** Biomarkers, Cardiology, Diseases, Medical research, Risk factors, Signs and symptoms

## Abstract

Traditionally the diagnosis of Metabolic syndrome (MetS) is binary (present/absent). The goal of this work is to propose a sex-specific continuous score to measure the severity of MetS in Mexican adults using waist circumference and body mass index as adiposity measures. MetSx-WC and MetSx-BMI indexes by sex were derived by confirmatory factor analysis (CFA) using data for 6567 adult participants of the National Health and Nutrition Survey 2018. The overall fit of the two proposed CFA models was excellent. We then validated these scores using a community-based health study of 862 university participants and determined that the reliability and strength of agreement between the MetSx-WC and MetSx-BMI scores were excellent. The ROC analysis of the resulting indexes indicates that they have excellent ability to discriminate a MetS classification according to the different criteria. The correlations of MetSx scores and surrogate markers of insulin resistance and obesity ranged from weak to strong. Subsequently, a retrospective study of 310 hospitalized patients with COVID-19 was used to determined that MetSx-BMI score was associated with the mortality of patients with COVID-19. The proposed indices provide a continuous measure in the identification of MetS risk in Mexican adults.

## Introduction

Metabolic syndrome (MetS) is a pathological condition characterized by abdominal obesity (AO), insulin resistance, hypertension, and hyperlipidemia^1^. It is considered a predictor of cardiovascular disease (CVD), type 2 diabetes mellitus (DM), and overall mortality^2^. The world is facing a growing epidemic of MetS. It is estimated that approximately one quarter of the world's adult population (1 billion) is affected by MetS^1^. In 2017, it was estimated that MetS affected 20% of the North American population, 25% of the European population, and approximately 15% of the Chinese population^2^. In Mexico, approximately 6 out of 10 Mexican adults have MetS (57.9% in men and 63.2% in women)^3^. A high prevalence of MetS has been reported among healthy Mexican adults, compared to reports from other countries, including the United States and Latin America^4^. Estimates of the prevalence of MetS have steadily increased in Mexico, mainly due to several multifactorial components in Mexicans, including genetic factors, an increase in the consumption of high-carbohydrate diets, a high prevalence of sedentary lifestyle, and limited use of public policies aimed at reducing the impact of MetS on the general population^5^.

A high prevalence of MetS has been reported among healthy Mexican adults, compared to reports from other countries, including the United States and Latin America^52^. Estimates of the prevalence of MetS have steadily increased in Mexico, mainly due to several multifactorial components in Mexicans, including genetic factors, an increase in the consumption of high-carbohydrate diets, a high prevalence of sedentary lifestyle, and limited use of public policies aimed at reducing the impact of MetS on the general population^53^. The most common component of MetS in Mexicans is AO and low HDL-cholesterol^3^. So, we need to develop indices to quantify the severity of MetS in the Mexican population.

MetS is traditionally presented as a binary classification (present/absent) based on criteria proposed by the National Cholesterol Education Program, Adult Treatment Program III (ATP-III); the American Association of the Heart/National Association of the Heart, Lung, and Blood Institute (AHA/NHLBI); and the International Diabetes Federation (IDF). Each criterion requires that a person have abnormalities in at least three components, but they have slightly different cut-off levels to designate an abnormality^6^. Gurka et Al. in the years 2012 and 2014 proposed a sex- and ethnicity-specific MetS for adolescents and adults in the US^7,8^. These indexes used the five traditional MetS components—waist circumference (WC), *high*-*density lipoprotein* *cholesterol* (HDL-c), systolic *blood pressure* (SBP), triglyceride (Tri), and glucose (Glu)—using data from the National Health and Nutrition Examination Survey (NHANES) for adolescents between 12 and 19 years^7^ and adults between 20 and 64 years^8^. These MetS indexes are highly correlated with other MetS surrogate markers, including highly sensitive C-reactive protein (hs-CRP), uric acid, and insulin resistance homeostasis model (HOMA-IR)^7,8^. Four years later, the same authors proposed another MetS index using body mass index (BMI) for adults instead of WC as a measure of adiposity, which provided similar power to predict future diseases as the previous index based on WC. Both indexes had a high degree of concordance^9^.

The most frequent components of MetS in the Mexican population are AO, low HDL-cholesterol, and high Tri levels^3^. OA and dyslipidemias separately or combined are the most prevalent MetSx components in the Mexican population, which expose this population to a high risk for diabetes^10,11^ and CVD^12^. In addition, hypertriglyceridemia in the Mexican population is higher than in other developing and developed countries, such as India, Nigeria, China, Japan, and the US^13^. Because the physiological and anthropometric characteristics of the Mexican population are different from those of the US population, it is necessary to have sex-specific indexes to measure the severity of MetS in Mexican adults.

The anthropometric indexes of general and central obesity most frequently used to assess the risk related to adiposity are BMI and WC^14^, because they can be easily and cheaply measured^15,16^. The BMI is the most common measure used in research and clinical practice^17^. WC has the best correlation with abdominal fat and the greatest association with cardiovascular risk factors, especially diabetes^18,19^. Hypertension and dyslipidemia have been shown to be associated with abnormal WC^20,21^. High BMI values (≥ 30 kg/${\mathrm{m}}^{2}$)^22^ are related to high risk of CVD^23,24^ and ischemic stroke^25^.

Motivated by the continuous scores of MetS developed by Gurka et al.^7–9^, we have the following goals: (1) propose specific MetS indexes for Mexican adults (MetSx) by gender using WC or BMI as a measure of adiposity; (2) evaluate the concordance between the two proposed indexes; (3) assess the association between the proposed indexes and various surrogate markers of insulin resistance and obesity as homeostatic model assessment for insulin resistance (HOMA-IR), lipid accumulation product (LAP), single-point insulin sensitivity estimator (SPISE), visceral adiposity index (VAI), triglyceride and glucose (TyG) index, and triglyceride/cholesterol-HDL ratio (TG/HDL), among others; and (4) examine the association between the MetSx-BMI score of hospitalized patients with COVID-19 and mortality outcomes.

## Results

### Derivation of equations of continuous score in ENSANUT

**Figure 1:**
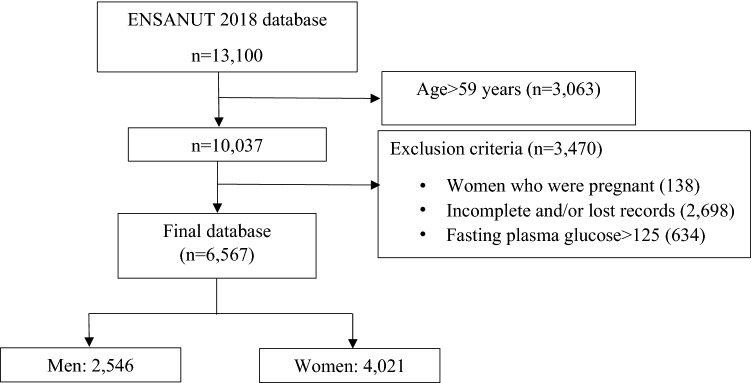
Flow diagram for obtaining the database for the formulation of the MetSx.

The ENSANUT database had 13,100 observations, but 3063 subjects older than 59 years were deleted. In addition, 3470 records were excluded according to the exclusion criteria (Fig. 1).

The final sample was composed of 6567 adults (2546 men and 4021 women). The average age was 37.90 (SD 11.41) years for men and 38.15 (SD 10.77) years for women. The descriptive statistics of the ENSANUT 2018 database are shown in Table 1. It should be noted that the average WC in both men and women is higher than the cut-off values proposed by the IDF and the harmonized criteria (HC). The BMI mean in both sexes indicates overweight (25.0 ≤ BMI ≤ 29.9). The mean systolic blood pressure (SPB) is higher in men than in women, the mean Glu is below the cut-off values proposed by the different criteria, and the mean Tri in women is close to the cut-off value of the different criteria (≥ 150 mg/dL).

**Table 1:** Characteristics of adults aged 20 to 59 years from ENSANUT 2018 about components of the metabolic syndrome and other biochemical parameters (n = 6567).

	Mean (SD)		
	Total sample	Women (n = 4021)	Men (n = 2546)
**Anthropometric**			
Age (year)	38.05 (11.02)	38.15 (10.77)	37.90 (11.41)
WC (cm)	94.06 (13.72)	93.33 (13.49)	95.21 (14.00)
BMI (kg/m^2^)	28.60 (5.75)	29.20 (5.93)	27.64 (5.31)
**Arterial blood pressure**			
Systolic BP (mmHg)	131.59 (39.63)	127.84 (37.70)	137.50 (34.89)
Diastolic BP (mmHg)	92.53 (48.45)	90.97 (48.54)	94.99 (48.21)
**Biochemical parameter**			
Glucose (mg/dL)	89.26 (11.68)	89.03 (11.58)	89.63 (11.64)
HbA1c (%)	5.19 (0.55)	5.23 (0.54)	5.15 (0.57)
Insulin (µUI/ml)	12.25 (13.71)	13.40 (14.72)	10.44 (11.72)
Triglycerides (mg/dL)	144.37 (53.51)	141.29 (52.79)	149.25 (54.29)
Cholesterol (mg/dL)	179.23 (35.56)	178.00 (35.03)	179.92 (36.39)
HDL cholesterol (mg/dL)	45.63 (10.73)	46.34 (10.44)	44.52 (11.07)
LDL cholesterol (mg/dL)	104.73 (30.21)	104.20 (29.40)	105.56 (31.43)
Creatinine (mg/dL)	0.71 (1.04)	0.62 (0.72)	0.85 (1.39)
Albumin (g/dL)	4.31 (0.88)	4.23 (0.89)	4.44 (0.85)
**Surrogate marker**			
HOMA-IR	2.80 (3.53)	3.05 (3.78)	2.41 (3.06)

The goodness-of-fit statistics and the AIC of the fitted models with the standardized variables are presented in Table 2. For each sex, both MetSx-WC and MetSx-BMI had RMSEA less than 0.08, which indicates good fitting. The rest of the indexes indicate excellent goodness of fit (AGFI, GFI, and CFI are all greater than 0.90, and SRMR is less than 0.08). The results of the GFI and AGFI are greater than the reference value 0.90 in the models. These indices account for the percentage of variance explained in the observed data and percentage of variance explained by the model, respectively.

**Table 2:** Goodness-of-fit indexes for the models in the CFA.

	MetSx-WC		MetSx-BMI		
	Women	Men	Women	Men	Ref
RMSEA	0.05	0.06	0.065	0.05	< 0.08
SRMR	0.03	0.03	0.03	0.03	< 0.08
AGFI	0.98	0.975	0.97	0.98	> 0.90
GFI	0.99	0.99	0.99	0.98	> 0.90
CFI	0.95	0.93	0.92	0.96	> 0.90
NFI	0.93	0.93	0.92	0.95	> 0.90
AIC	56,039	35,630	56,026	35,448	

We report the standardized factor loadings to MetSx-WC and MetSx-BMI in Table 3. SBPZ's factor loadings in both men and women were close to 0.23. lnTriZ's factor loadings had the highest values compared to the other MetS components, and women had higher factor loads than men. Men had higher factor loadings of HDLNZ than women; in contrast, GluZ's factor loadings are higher in women than in men.

**Table 3:** Confirmatory factor analysis estimation with transformed and standardized variables.

	*WCZ*			*BMIZ*		
	Women	Men	p	Women	Men	p
	SFW	SFW		SFW	SFW	
Measure of obesity	1.000	1.000		1.000	1.000	
*HDLNZ*	0.658	0.785	** < 0.01**	0.661	0.771	** < 0.01**
*lnTriZ*	0.946	0.898	** < 0.01**	0.895	0.860	** < 0.01**
*SBPZ*	0.229	0.235	** < 0.01**	0.233	0.213	** < 0.01**
*GluZ*	0.691	0.503	** < 0.01**	0.697	0.485	** < 0.01**

The letter N in HDL indicates that the negative of HDL was used, and the letter Z indicates that the variable was standardized. SFW: Standardized factorial weights. Significant p-values are in bold.

Table 4 provides equations to calculate the MetSx severity score by sex for the Mexican adult population using WC or BMI. Although the analysis was performed using transformed variables, these indexes are given in the original scales of the MetSx variables used.

**Table 4:** MetSx by sex for Mexican adults.

**Using WC**
Women
$$-21.60+0.074\cdot WC-0.063\cdot \mathrm{HDL}+2.365\cdot lnTri+0.006\cdot SPB+0.060\cdot Glu$$
Men
$$-19.447+0.071\cdot WC-0.070\cdot \mathrm{HDL}+2.245\cdot lnTri+0.006\cdot SPB+0.042\cdot Glu$$
**Using BMI**
Women
$$-19.052+0.168\cdot BMI-0.063\cdot \mathrm{HDL}+2.237\cdot lnTri+0.006\cdot SPB+0.060\cdot Glu$$
Men
$$-17.20+0.188\cdot BMI-0.069\cdot \mathrm{HDL}+2.150\cdot lnTri+0.006\cdot SBP+0.041\cdot Glu$$

### Validation of the proposed scores in CBHS

The characteristics by sex of the participants of the validation dataset are presented in **S**upplemental Table 1. Men have a higher WC than women, as well as BMI. Biochemical parameters are similar in both men and women. The participants of CBHS have different characteristics (anthropometrics, arterial blood pressures, and biochemical parameters) compared to the participants of ENSANUT 2018.

The prevalence of MetSx based on the four diagnostic criteria was considerably higher in women (Table 5) in the CBHS database. According to the HC, 44.38% of women and 28.90% of men had MetSx.

**Table 5:** Prevalence of metabolic syndrome according to the different criteria.

Criteria	Women n (%)	Men n (%)
ATP III	94 (16.75)	38 (12.62)
AHA/NHLBI	202 (36.00)	46 (15.28)
IDF	235 (41.88)	82 (27.24)
IDF harmonized	249 (44.38)	87 (28.90)

In Table 6, we show the performance of the proposed indexes with different surrogate markers of insulin resistance and obesity. MetSx values correlated strongly with TyG index in both men and women. SPISE, METS-IR, TG/HDL ratio, LAP, and VAI moderately correlated with both MetSx in women, but these are strongly correlated in men. Both MetSx have a weak correlation with uric acid, METS-VF, and VAT and a null correlation with creatinine.

**Table 6:** Correlations (95% CI) of biochemical parameters and surrogate markers with the MetSx.

	Women		Men	
	MetSx-WC	MetSx-BMI	MetSx-WC	MetSx-BMI
**Biochemical parameter**				
Uric acid	0.11 (0.03, 0.19)	0.11 (0.02, 0.18)	−0.05 (−0.16, 0.05)	−0.07 (−0.18, 0.04)
Creatinine	−0.01 (−0.09, 0.07)	−0.02 (−0.10, 0.06)	−0.08 (−0.19, 0.03)	−0.08 (−0.19, 0.03)
HbA1c (%)	0.72 (0.68, 0.76)	0.72 (0.68, 0.76)	−0.14 (−0.25, −0.03)	−0.14 (−0.25, −0.23)
**Surrogate marker**				
ln(HOMA-IR)	0.59 (0.53, 0.64)	0.59 (0.53, 0.64)	0.01 (−0.10, 0.12)	0.01 (−0.10, 0.12)
QUICKI	−0.57 (−0.62, −0.51)	−0.56 (−0.61, −0.51)	−0.23 (−0.33, −0.12)	−0.23 (−0.33, −0.12)
SPISE	−0.57 (−0.62, −0.51)	−0.60 (−0.65, −0.54)	−0.72 (−0.77, −0.66)	−0.78 (−0.82, −0.73)
METS-IR	0.60 (0.54, 0.65)	0.62 (0.57, 0.67)	0.72 (0.66, 0.77)	0.78 (0.73, 0.82)
TyG	0.83 (0.80, 0.85)	0.83 (0.80, 0.85)	0.87 (0.84, 0.89)	0.86 (0.82, 0.88)
TG/HDL ratio	0.55 (0.50, 0.61)	0.55 (0.50, 0.61)	0.73 (0.67, 0.78)	0.72 (0.66, 0.77)
LAP	0.62 (0.57, 0.67)	0.58 (0.52, 0.62)	0.77 (0.73, 0.81)	0.75 (0.70, 0.80)
VAI	0.56 (0.50, 0.61)	0.53 (0.47, 0.59)	0.74 (0.69, 0.79)	0.72 (0.66, 0.77)
METS-VF	0.36 (0.29, 0.43)	0.34 (0.26, 0.41)	0.47 (0.38, 0.55)	0.48 (0.39, 0.56)
VAT	0.39 (0.32, 0.46)	0.37 (0.29, 0.44)	0.47 (0.38, 0.55)	0.48 (0.39, 0.56)

The ICC values indicate a high degree of concordance between the two MetSx by sex; they are 0.982 and 0.975 for women and men, respectively. On the other hand, the CCC values also show excellent agreement between the two MetSx they are 0.982 and 0.975 for women and men, respectively (Table 7).

**Table 7:** Concordance and intraclass correlation coefficients between MetSx-WC and MetSx-BMI.

	MetSx-WC			
	ICC (95% CI)		CCC (95% CI)	
	Women	Men	Women	Men
MetSx-BMI	0.982 (0.979, 0.985)	0.975 (0.969, 0.980)	0.982 (0.979, 0.985)	0.975 (0.970, 0.980)

*ICC* intraclass correlation coefficient, *CCC* concordance correlation coefficient, *CI* confidence interval.

ROC analysis of the formulated risk scores in our CBHS database displays an excellent ability to predict a MetS binary classification with the defined operational definitions (minimum AUC values of 0.875 and 0.904 for men and women, respectively). MetSx-WC had the largest AUC for both sexes and whatever criteria. In females, the AUC was lower in all cases for MetSx-BMI, and in men the lower AUC varied according to the four most important MetS classification criteria (Table 8).

**Table 8:** AUC of MetSx for the binary diagnosis of MetS.

Index	Women			Men		
	AUC	95% CI	p	AUC	95% CI	p
**ATP III**						
MetSx-WC	0.930	(0.908, 0.953)	0.30	0.934	(0.902, 0.966)	0.58
MetSx-BMI	0.925	(0.901, 0.948)		0.932	(0.900, 0.964)	
**AHA/NHLBI**						
MetSx-WC	0.935	(0.914, 0.956)	** < 0.01**	0.925	(0.885, 0.966)	0.69
MetSx-BMI	0.918	(0.895, 0.941)		0.923	(0.886, 0.963)	
**IDF**						
MetSx-WC	0.918	(0.895, 0.941)	** < 0.01**	0.884	(0.844, 0.923)	0.20
MetSx-BMI	0.904	(0.880, 0.927)		0.875	(0.834, 0.916)	
**IDF harmonized**						
MetSx-WC	0.939	(0.921, 0.958)	** < 0.01**	0.902	(0.866, 0.938)	0.20
MetSx-BMI	0.929	(0.907, 0.949)		0.894	(0.856, 0.931)	

*AUC* area under the ROC curve, *CI* confidence interval.

Significant p-values are in bold.

The AUCs of the MetSx-BMI and MetSx-WC (See Supplemental Fig. 1) are statistically equal in men to the MetS diagnostic criteria (p > 0.05). Under the ATP-III criterion, the AUCs of the MetSx-IMC and MetSx-WC (See Supplemental Fig. 2) are statistically equal in women (p > 0.05), whereas with the other criteria, MetSx-WC presents higher AUCs (p < 0.05).

### Application of MetSx-BMI score in COVID-19

In total, 310 patients (77.7% males), with a mean age of 44.78 (SD 8.61) years, were included in this retrospective study. The characteristics of MetSx of the survivors and non-survivors are shown in Supplemental Table 2.

A comparative and adjusted Cox model analyses are shown in Table 9. There is a median statistically significant difference in the MetSx-BMI score between non-survivor and survivor groups. The median of MetSx-BMI score among non-survivors was 5.247(Q1: 2.944, Q3: 7.216), and quartiles measures are above quartile 3 of MetSx-BMI score distributions by ENSANUT 2018 (Supplemental Fig. 4). In summary, age (adjusted HR 1.039 95% IC 1.015–1.065, p = 0.002) and MetSx-BMI score (adjusted HR 1.075 95% IC 1.037–1.114, p < 0.001) were associated with a significantly increased risk of death by COVID-19. Sex was not a risk factor 1.006 (adjusted HR = 95% IC 0.626–1.617, p = 0.979). The anticoagulant therapy was associated with a significantly decreased risk of death by COVID-19, 0.236 (adjusted HR = 95% IC 0.102–0.548, p = 0.001). Linezolid therapy was not statistically significant 0.990 (adjusted HR = 95% IC 0.590–1.662, p = 0.969).

**Table 9:** Risk factors associated with mortality in patients hospitalized with COVID-19.

Characteristics	Outcome			Cox proportional-hazards model	
	Non-survivor (n = 101)	Survivor (n = 209)	p	Adjusted HR (95% CI)	p
Sex (men)	77 (76.2%)	164 (78.5%)	0.658*	1.006 (0.626,1.617)	**0.979**
Age (years)	47.89 (7.90)	43.28 (8.56)	** < 0.001**^**†**^	1.039 (1.015,1.065)	**0.002**
MetSx-BMI	5.247(2.944,7.216)^+^	2.045(0.670,3.778)^+^	** < 0.001**^**‡**^	1.075 (1.037,1.114)	** < 0.001**
Anticoagulant therapy (yes)	6 (5.9%)	42 (20.1%)	**0.001**	0.236 (0.102,0.548)	**0.001**
Linezolid therapy (yes)	20 (19.8%)	38 (18.2%)	0.732	0.990 (0.590–1.662)	0.969

*HR* hazard ratio, *CI* confidence interval.

^+^Quartile 2(Quartile 1, Quartile 3).

*Chi-squared test.

^†^t-test.

^‡^Median test.

Significant p-values are in bold.

## Discussion

The present study shows sex differences among adults in the standardized factorial weights (SFW) of the Mets (Table 3), resulting in moderate or strong associations of MetSx scores with subrogated markers of insulin resistance and obesity. We observed that in the MetSx scores for women, the SFW of the obesity measure (BMI and WC), Tri, and Glu have the greatest relative weight on the MetSx-BMI and MetSx-BMI scores. On the other hand, in the scores for men, the components with the greatest relative weight are obesity measure, Tri, and HDL. This sexual dimorphism between the MetS components has been reported in the scores developed by Gurka et al.^7–9^. In the Hispanic ethnic group in the American population, the components with the greatest relative weight in the score are, in decreasing order, Tri, HDL, obesity measure, Glu, and SPB in women, and Tri, obesity measure, HDL, Glu, and SPB in men^9^. In contrast, in the Mexican population, the MetS components with greater relative weight in both MetSx-BMI and MetSx-BMI scores were obesity measure and Tri, followed by Glu, HDL, and SPB in women and by HDL, Glu, and SPB in men. Clearly, our findings are consistent with those reported in the Mexican population that AO and dyslipidemia are the common MetS components^3^.

In the university population, we find that the (known and novel) surrogate markers of insulin resistance and obesity have a moderate to strong correlation with the MetSx scores. TyG index, SPISE, METS-IR, and TG/HDL ratio have no weak association with MetSx scores, which shows the association of insulin resistance and MetS. Many authors have identified LAP, VAI, and TyG index as the best in performance for identifying MetS cases by using binary MetS criteria^54–56^. Gurka et al. reported a moderate correlation of scores with ln(HOMA-IR)^8,57^. In the university population, women showed a moderate association of ln(HOMA-IR) with MetSx scores, but there was a null association in men. LAP is an index used for evaluating lipid overaccumulation in adults^35^. We find a moderate and strong association in women and men with both MetSx scores. Rotter et al.^58^ reported associations between LAP and MetS and its components in aging men. Amato et al.^36^ derived and associated the VAI with cardiometabolic risk. We found a moderate to strong association of VAI with MetSx scores. Previously, Wang et al.^59^ found a longitudinal association of MetSx scores with biomarker of oxidative stress leading to CVD.

Using the CBHS dataset, very different estimated prevalences of MetS were found using the four identification criteria. The lowest estimated prevalence is obtained with the ATP III criterion, followed by that obtained with the AHA/NHLBI criterion, then with the IDF criterion, and the highest prevalence is obtained with IDF-harmonized; the latter two were notably higher (Table 5). With all criteria, the estimated prevalence of MetS is higher for women than for men. In women, the prevalence was higher according to the HC of the IDF and the IDF. These variations between the prevalences of the MetS criteria have already been reported by Espósito et al.^60^, where ATPIII criteria identify a lower number of MetS cases compared to the other criteria. Also, it shows an excellent reliability and degree of agreement between the two scores by sex. A similar result was found by Gurka et al.^9^ when validating the interchangeability of the obesity measures in the score derivation. The MetSx scores showed an almost perfect ability to discriminate a classification based on the different MetS criteria (AUC > 0.875). The highest AUC is found by the indexes under the HC in women (Supplemental Fig. 2). In men, the highest AUC is observed by the indexes under the ATP III criteria (Supplemental Fig. 1).

The Cox proportional-hazard regression found that age and MetsSx-IBM score were associated with the mortality of patients hospitalized with COVID-19. Our results match with other studies that found age as one of the most important factors in COVID-19 severity (59). Many of the parameters considered by the MetSx-BMI score have been associated with a more severe course of COVID-19, including obesity, overweight, low levels of HDL-c, and hypertriglyceridemia^60–62^. Furthermore, MetS is emerging as an important characteristic in young adults with complications from COVID-19. The increased mortality risk of patients with MetS could be associated with the enhanced expression of ACE2, IL6, and TNF- α adipose tissue secretion and elevated concentration of von Willebrand factor and plasminogen activator inhibitor-1^63^. In short, it seems that the proinflammatory state in MetS could overlap with the acute inflammatory state, increasing the probability of cytokine storm, a pathological situation related with organ damage and death in COVID-19.

Our study has some limitations. We use the ENSANUT 2018 database, which is obtained with a complex probabilistic sampling design of all Mexican states. Although the methodology is robust, it is transversal. These scores should be used in the future to provide a continuous measure to track changes in MetS-related abnormalities in each subject over time. Another limitation is that the scores could only be applied to the adult Mexican population. Determination of the different cut-off values for risky thresholds associated with different diseases remains to be done. The university population of the CBHS could have selection bias. Finally, the RSCOVID-19 database of patients hospitalized with COVID-19 is a retrospective study.

In conclusion, we proposed sex-specific continuous score to measure the severity of MetS in Mexican adults using WC and BMI as adiposity measures. The proposed indices assume that MetS is a single latent factor and that the sum of the components of MetS does not have the same weight. It is important to bear in mind that our goal was not to explore prevalence nor to study the factors of MetS in the Mexican population. MetSx-WC and MetSx-BMI scores can be important tools for clinical use, such as risk identification and appropriate patient monitoring over time. They could be used to quantify the degree of severity associated with diabetes, CVD, chronic kidney disease, metabolic dysfunction-dissociated fatty liver disease (MAFLD), advanced fibrosis, and numerous malignancies in Mexican populations. In a future study, we will determine the MetSx-BMI score cut-off value for mortality from COVID-19.

## Methods

### ENSANUT and score derivation

Data were obtained from the México’s National Health and Nutrition Survey 2018 (ENSANUT 2018). The methodology, design details, and sample size determination are described by the document prepared jointly by the Mexican National Institute of Public Health (Spanish acronym: INSP) and the National Institute of Statistics and Geography Health (Spanish acronym: INEGI)^26,27^. Anthropometric measurements were taken, as well as blood pressure. The peripheral blood biological sample collection was performed using calibrated equipment^27^. For the present study, inclusion criteria were Mexican men or women between 20 and 59 years of age, and exclusion criteria were incomplete and/or lost records, pregnant women and participants with known or unknown diabetes (fasting plasma Glu>125mg/dL), since an unbiased setting of metabolic disorder is sought, and all these conditions are likely to alter lipid and insulin levels^7–9^.

BMI was calculated using the following formula:

$$BMI=\frac{Weight(kg)}{{(Height(m))}^{2}}$$

Several confirmatory factor analyses (CFAs) were performed, having in common four MetS components in adults (SBP, HDL-c, Tri, and Glu), but differing in the inclusion of one variable that measures adiposity, WC (MetSx-WC) or BMI (MetSx-BMI). Following Gurka et al.^7–9^, Tri were natural log-transformed (*lnTri*) to fit the normal distribution, and the negative of HDL-c (HDLN) was used so a higher factor loading score would be similar in interpretation to the other measures in the model. After that, all variables were standardized (mean = 0 and standard deviation = 1) (*WCZ*, *BMIZ*, *GluZ*, *lnTriZ*, *HDLNZ*, *SBPZ*). The factor loadings (λ) for each of the measured variables indicate the strength of the association between the variables and the MetSx factor. Parameter estimation was performed by maximum likelihood. The equations that define the MetSx index were obtained by inverse transformation of the coefficients that resulted from the CFA with the standardized variables. That is, MetSx equations use the original measurements of the variables to facilitate clinical use.

The quality of the fitted CFA models was assessed using different goodness-of-fit statistics, such as the Bentler–Bonett normed fit index (NFI; poor fit < 0.90), comparative fit index (CFI; poor fit < 0.90), root mean square error of approximation (RMSEA; poor fit > 0.06), standardized root mean squared residual (SRMR; poor fit > 0.08), goodness of fit index (GFI; poor fit < 0.90), and adjusted goodness of fit index (AGFI; poor fit < 0.90)^32^. In addition, the Akaike's information criterion (AIC) was used for model comparison, where a small AIC value indicates a better fit.

### Validation: community-based health study (CBHS)

Data from a cross-sectional study of community health in a population composed of students, academics, and support staff of the National Autonomous University of Mexico (Spanish acronym: UNAM) were used to validate the proposed indexes and to find their correlation with different surrogate markers of insulin resistance and obesity. Data include sociodemographic information, anthropometric measurements, blood pressure measurement, and biochemical parameters determined by the laboratory results of an 8-h fast^33^.

The presence of MetS was declared using the three criteria most important and the IDF’s harmonized criteria *(HC)*. ATP III declares the presence of MetS when three or more of the following abnormalities occur: WC ≥ 102 cm in men, ≥ 88 cm in women; Glu ≥ 110 mg/dL or previous diagnosis of diabetes; Tri ≥ 150 mg/dL; SBP ≥ 130 mm Hg or previous diagnosis of hypertension; HDL-c < 40 mg/dL in men and < 50 mg/dL in women^28^. AHA/NHLBI declares the presence of MetS when three or more of the following abnormalities occur: WC ≥ 102 cm in men, ≥ 88 cm in women; Glu ≥ 100 mg/dL or previous diagnosis of diabetes; Tri ≥ 150 mg/dl or drug treatment to control Tri; SBP ≥ 130 mm Hg or previous diagnosis of hypertension; HDL-c < 40 mg/dl in men or < 50 mg/dl in women^29^. The IDF declares the presence of MetS when three or more of the following abnormalities occur: WC ≥ 90 cm in men or ≥ 80 cm in women plus two or more of the following conditions: Tri ≥ 150 mg/dL or drug treatment to control Tri; HDL-c < 40 mg/dL in men and < 50 mg/dL in women; SBP ≥ 130 mm Hg or previous diagnosis of hypertension; Glu ≥ 100 mg/dL or previous diagnosis of diabetes^30^. Finally, in the HC, at least three of the five following components mean the presence of MetS^31^: WC in men ≥ 90 cm and in women ≥ 80 cm, hyperglycemia (Glu ≥ 100 mg/dl or drug treatment for Glu control), hypertriglyceridemia (Tri ≥ 150 mg/dL), HDL-c in men < 40 mg/dL and in women < 50 mg/dL, or elevated blood pressure (SBP ≥ 130 mm Hg or drug treatment to control hypertension).

Agreement between the MetSx-WC and MetSx-BMI indexes was assessed using the intraclass correlation coefficient (ICC) and concordance correlation coefficient (CCC)^43,44^, where ICC values below 0.5 indicate poor reliability, values between 0.5 and 0.75 indicate moderate reliability, values between 0.75 and 0.9 indicate good reliability, and values above 0.90 indicate excellent reliability. Mcbride^44^, using CCC, classifies the strength of agreement as poor (< 0.90), moderate (0.90–0.95), substantial (0.95–0.99), and near perfect (> 0.99). Receiver operator characteristic (ROC) curve was used to measure the discriminatory ability of the indexes over the range of possible values in the detection of MetS quantified by the area under the curve (AUC). AUCs were compared using the nonparametric approach of DeLong et al.^45,46^.

Known and novel surrogate markers of insulin resistance and obesity are HOMA-IR, LAP, SPISE, VAI, TyG, TG/HDL, metabolic score for insulin resistance (METS-IR), metabolic score for visceral fat (METS-VF), quantitative insulin sensitivity index (QUICKI), and intra-abdominal and visceral fat (VAT). They were calculated using the following formulas^33–41^:

- $$\mathrm{HOMA}-\mathrm{IR}=\frac{\mathrm{INS}\left(\mathrm{\mu UI}/\mathrm{L}\right)\times \mathrm{ Glu }(\mathrm{mg}/\mathrm{dL}) }{18\times 22.5}$$
- $$\mathrm{SPISE}=\frac{600\times {\mathrm{HDL}(\mathrm{mg}/\mathrm{dL})}^{0.185}}{{\mathrm{Tri}(\mathrm{mg}/\mathrm{dL})}^{0.2}{\times \mathrm{BMI}}^{1.338}}$$
- $$\mathrm{QUICKI}={\left\{\mathrm{ln}[\mathrm{INS }(\mathrm{\mu UI}/\mathrm{L})]+\mathrm{ln }[\mathrm{Glu }(\mathrm{mg}/\mathrm{dL})]\right\}}^{-1}$$
- $$\mathrm{METS}-\mathrm{IR}=\frac{{\text{ln}}[2\times \text{ Glu}(\mathrm{mg}/\mathrm{dL})+\text{ Tri}(\mathrm{mg}/\mathrm{dL})]\times \mathrm{BMI}\left(\mathrm{kg}/{\mathrm{m}}^{2}\right)}{{\text{ln}}[\mathrm{HDL}(\mathrm{mg}/\mathrm{dL})]}$$
- $$\mathrm{TG}/\mathrm{HDL}=\frac{\mathrm{Tri}(\mathrm{mg}/\mathrm{dL})}{\mathrm{HDL}(\mathrm{mg}/\mathrm{dL})}$$
- $$\mathrm{TyG}=\frac{\mathrm{ln}\left(\mathrm{Tri}(\mathrm{mg}/\mathrm{dL})\times \mathrm{Glu}(\mathrm{mg}/\mathrm{dL})\right)}{2}$$
- $${\mathrm{LAP}}_{\mathrm{Women}}=\left(\mathrm{WC}(\mathrm{cm})-58\right)\times \mathrm{Tri}(\mathrm{mmol}/\mathrm{L})$$
- $${\mathrm{LAP}}_{\mathrm{Man}}=\left(\mathrm{WC}(\mathrm{cm})-65\right)\times \mathrm{Tri}(\mathrm{mmol}/\mathrm{L})$$
- $${\mathrm{VAI}}_{\mathrm{Women}}=\left(\frac{\mathrm{WC}(\mathrm{cm})}{\left(1.89\times \mathrm{BMI}\left(\mathrm{kg}/{\mathrm{m}}^{2}\right)\right)+36.58}\right)\times \left(\frac{\mathrm{Tri}(\mathrm{mmol}/\mathrm{L})}{0.81}\right)\times \left(\frac{1.52}{\mathrm{HDL}(\mathrm{mmol}/\mathrm{L})}\right)$$
- $${\mathrm{VAI}}_{\mathrm{Man}}=\left(\frac{\mathrm{WC}(\mathrm{cm})}{\left(1.88\times \mathrm{BMI}\right)+39.68}\right)\times \left(\frac{\mathrm{Tri}(\mathrm{mmol}/\mathrm{L})}{1.03}\right)\times \left(\frac{1.31}{\mathrm{HDL}(\mathrm{mmol}/\mathrm{L})}\right)$$
- $$\begin{aligned}\text{METS }-\mathrm{VF}& = 4.466+0.011({\text{ln}}(\text{ METS}-\mathrm{IR}){)}^{3} +3.239({\text{ln}}(\mathrm{WHtR}){)}^{3}\\ &\quad + 0.319\left(\mathrm{Sex}\right)+0.594 {\text{ln}}(\text{Age })\end{aligned}$$
- $$\begin{array}{cc}{\text{VAT}}& ={\mathrm{e}}^{4.466+0.011\left[(\mathrm{ln}(\mathrm{METS}-\mathrm{IR}){)}^{3}\right]+3.239\left[(\mathrm{ln}(\mathrm{WHtR}){)}^{3}\right]+0.319(\mathrm{Sex})+0.594({\text{ln}}({\text{Age}}))}\\ & \end{array}$$

where Sex = 1(0) if man (woman), and $\mathrm{WHtR}=\frac{\mathrm{WC}}{\mathrm{height}}$ denotes waist-to-height ratio.

Finally, for CBHS, Pearson's linear correlation was used to measure the association between insulin surrogate and obesity markers and the MetSx indexes by sex proposed, where values from 0 to 0.3 (or 0 to −0.3) are insignificant; 0.3 to 0.5 (or −0.3 to −0.5) are weak; 0.5 to 0.7 (or −0.5 and −0.7) are moderate; 0.7 to 0.9 (or −0.7 to −0.9) are strong; and correlations > 0.9 (or < −0.9) are very strong^42^.

### Application: relation between MetSx-BMI score and COVID-19

To examine the relationship between MetSx-BMI score and COVID-19 mortality, data from a retrospective study with hospitalized patients (RSCOVID-19) were used. The participants were confirmed to have SARS-CoV-2 infection in a tertiary referral hospital in Mexico City, Mexico (Central Military Hospital [Spanish acronym: HCM], from the National Secretariat of Defense [Spanish acronym: SEDENA]). SARS-CoV-2 infection was confirmed using real-time reverse transcription polymerase chain reaction from a nasopharyngeal swab. Epidemiological data including age, sex, BMI, blood pressure measurement, and biochemical parameters were obtained from electronic health records.

A multivariate Cox proportional hazard regression was performed to identify explanatory variables associated with SARS-CoV-2 death during hospitalization. Hazard ratios (HRs) were estimated as a measure of size effect of the variables included in the Cox regression. The proportional hazard assumption was evaluated with the Schoenfeld residuals method test. Statistical analysis was performed in R software with a significance level of 0.05, using the packages *lavaan*^47^, *DescTools*^48^, *irr*^49^, *pROC*^50^, and *survival*^51^.

### Ethical considerations

This study is based on an analysis of two public free-access databases (ENSANUT 2018 and CBHS) and one restricted database (RSCOVID-19). ENSANUT 2018 protocols have the approval of the Ethical and Research Commissions of the National Institute of Public Health. CBHS was approved by the Ethics Committee of the Faculty of Medicine of the National Autonomous University of Mexico, and data are available in the UNAM repository at http://www.c3.unam.mx/health/. RSCOVID-19 was approved (registration no. 066/2020) by the research committee at Central Military Hospital, and we obtained the permissions to access and use data from the restricted database RSCOVID-19. ENSANUT 2018 and CBHS were explained, and a written informed consent was obtained from all participants. In RSCOVID-19 study we used a de-identified version of this dataset, with the assent of the research committee at CMH to process patient confidential data without explicit patient consent. All methods were carried out in accordance with relevant guidelines and regulations.

## Supplementary Information

Supplementary Information.

## Data Availability

ENSANUT 2018 and CBHS are public free-access databases. The RSCOVID-19 database is available upon reasonable request to the corresponding author.
