# Supplementary material for: Derivation and validation of sex-specific continuous metabolic syndrome scores for the Mexican adult population
Source: Sci Rep. 2022 Jun 10;12:9659. doi: 10.1038/s41598-022-10963-w (PMC9187334; doi:10.1038/s41598-022-10963-w)
Supplement: Supplementary file 1 — Supplementary Information. [file 41598_2022_10963_MOESM1_ESM.docx]

**Supplementary Information**

In Table 1, the ENSANUT 2018 results showed gender difference in the MetS compounds. The main differences were higher BMI, insulin levels, and HOMA-IR score for women. There is evidence that indicates that women are more likely to have a more insulin-sensitive environment than men, probably because of the distribution of adipose tissue and the difference of sex hormones between the sexes [S1]. However, in the case of México, many social and race factors resulted in higher prevalence of obesity and insulin resistance in women compared to men. It is estimated that 40% of the women in Mexico are obese (BMI >30), while the prevalence of obesity in men is 30% [S2]. Another possible explanation of the results observed in HOMA-IR is that this biomarker follows a nonlinear correlation with age in women with an increase in levels after 50 years of age [S3].

Supplemental Table 1: Descriptive statistics of the CBHS database for validation of MetSx

| Parameters | Mean (SD) | |
| --- | --- | --- |
|  | Women (n=561) | Men  (n=301) |
| Anthropometric |  |  |
| Age (years) | 41.13 (10.05) | 39.92 (10.62) |
| WC (cm) | 90.34 (12.10) | 95.55 (12.12) |
| BMI (kg/m^2^) | 26.69 (4.94) | 26.96 (4.79) |
| Arterial blood pressure |  |  |
| Systolic BP (mmHg) | 110.12 (14.11) | 115.12 (14.48) |
| Diastolic BP (mmHg) | 72.41 (10.56) | 75.75 (10.89) |
| Biochemical parameter |  |  |
| Glucose (mg/dL) | 99.21 (36.32) | 95.53 (26.36) |
| HbA1c (%) | 5.42 (1.37) | 5.32 (1.07) |
| Insulin (µUI/ml) | 8.52 (6.02) | 8.40 (6.48) |
| Triglycerides (mg/dL) | 169.10 (99.13) | 167.63 (116.55) |
| Cholesterol (mg/dL) | 204.65 (36.50) | 205.19 (40.63) |
| HDL cholesterol (mg/dL) | 47.11 (11.64) | 48.28 (13.00) |
| LDL cholesterol (mg/dL) | 123.17 (32.51) | 122.47 (33.74) |
| Creatinine (mg/dL) | 0.83 (0.55) | 0.78 (0.18) |
| Uric acid (mg/dL) | 5.38 (1.47) | 5.37 (1.42) |
| Surrogate marker |  |  |
| HOMA-IR | 2.18 (2.24) | 2.07 (1.88) |
| QUICKI | 0.15 (0.02) | 0.16 (0.02) |
| SPISE | 5.87 (1.557 | 5.85 (1.58) |
| METS-IR | 41.10 (8.65) | 41.20 (8.72) |
| TyG | 4.78 (0.31) | 4.74 (0.33) |
| TG/HDL | 3.97 (2.93) | 4.02 (3.61) |
| LAP | 62.81 (45.35) | 58.55 (47.22) |
| VAI | 3.37 (2.52) | 2.36 (2.12) |
| METS-VF | 6.55 (0.56) | 6.79 (0.54) |
| VAT | 803.83 (400.3) | 1011.75 (498.4) |

The CBHS results in Supplemental Table 1 are similar to the results of the ENSANUT 2018 in the higher levels of arterial blood pressure among men and similar results of lipid profile between sexes. Moreover, the biomarkers associated with insulin resistance like insulin blood levels and HOMA-IR were higher in women. The BMIs were similar between sexes, which could suggest that insulin resistance in the Mexican population has more physiological causes despite obesity and overweight. Multiple situations, including genetics, lifestyle, and acculturation, have been found in the higher risk of Hispanic women for obesity [S4].


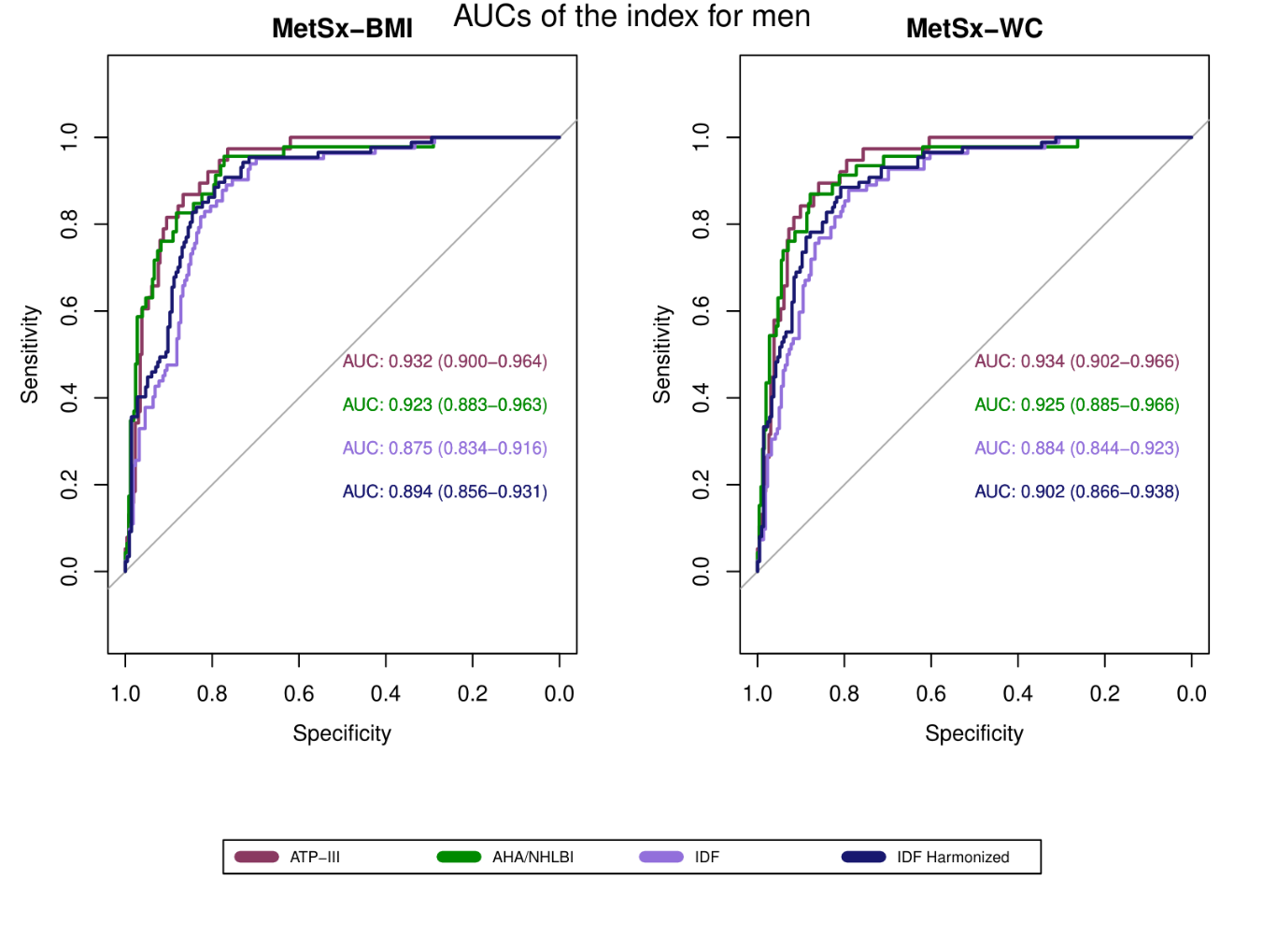
Supplemental Figure 1. AUCS of MetSx-WC and MetSx-BMI indexes for men by MetS classification criteria.


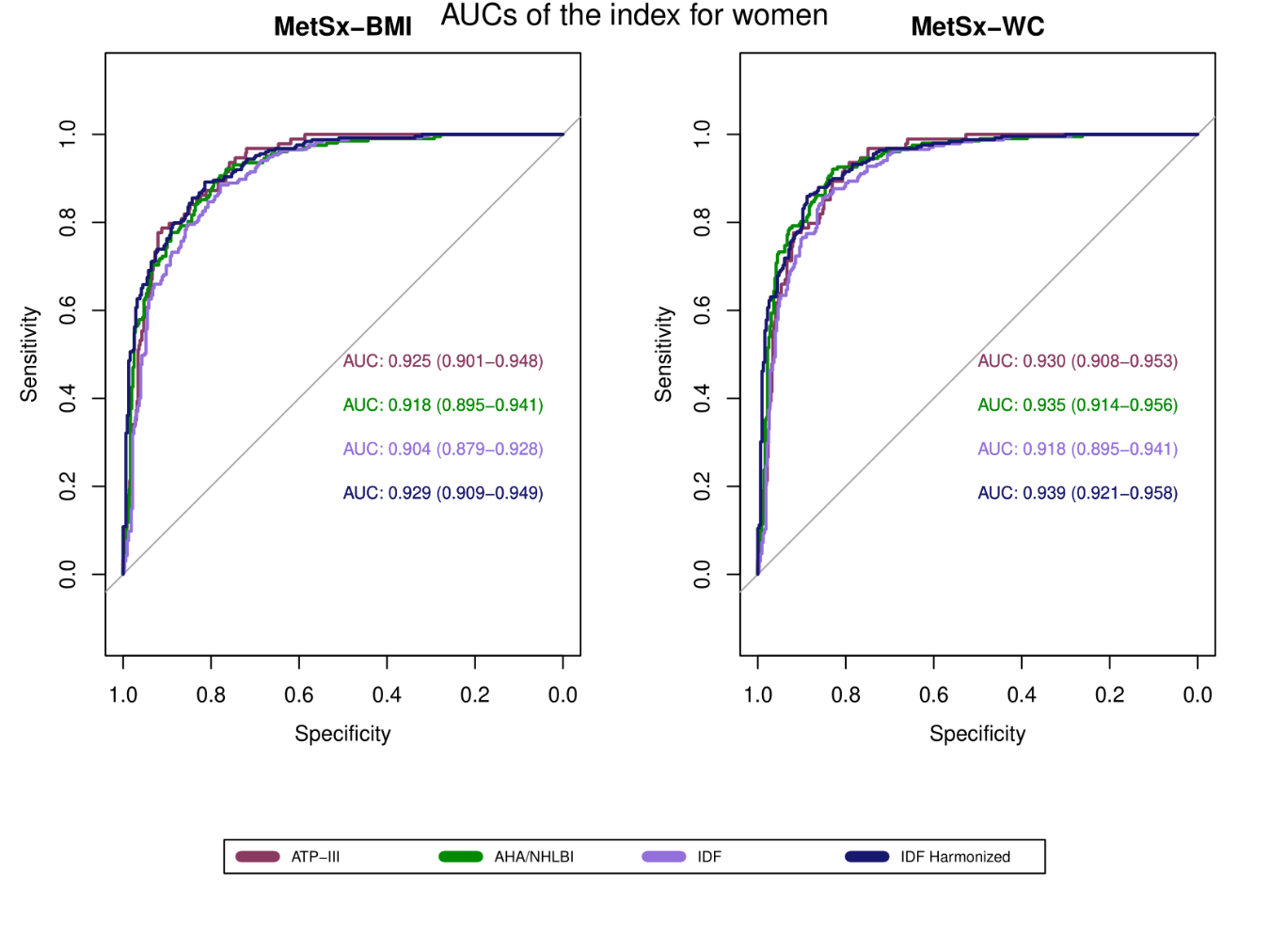


Supplemental Figure 2. AUCS of MetSx-WC and MetSx-BMI indexes for women by MetS classification criteria.

Supplemental Table 2 shows the difference in the parameters in RSCOVID-19 among survivors and non-survivors after hospitalization with COVID-19 in a third-level hospital. Non-survivors had higher levels of Glu, triglycerides, and HDL-C. This suggests that insulin resistance-associated parameters could have an important role in the risk of a patient to develop a more severe clinical disease course with COVID-19.

Supplemental Table 2: Descriptive statistics of the survivors of COVID-19 for application of MetSx

| Parameters | Mean (SD) | |
| --- | --- | --- |
|  | Non-survivors  (n=101) | Survivors  (n=209) |
| Anthropometric |  |  |
| BMI (kg/m^2^) | 27.65 (4.81) | 27.36 (4.60) |
| Arterial blood pressure |  |  |
| Systolic BP (mmHg) | 125.45 (37.92) | 122.19 (14.51) |
| Diastolic BP (mmHg) | 71.79 (13.49) | 75.86 (10.96) |
| Biochemical parameter |  |  |
| Glucose (mg/dL) | 155.93 (82.00) | 125.98 (80.70) |
| Triglycerides (mg/dL) | 356.40 (288.09) | 213.74 (143.71) |
| HDL cholesterol (mg/dL) | 22.29 (9.28) | 31.91 (18.13) |

| 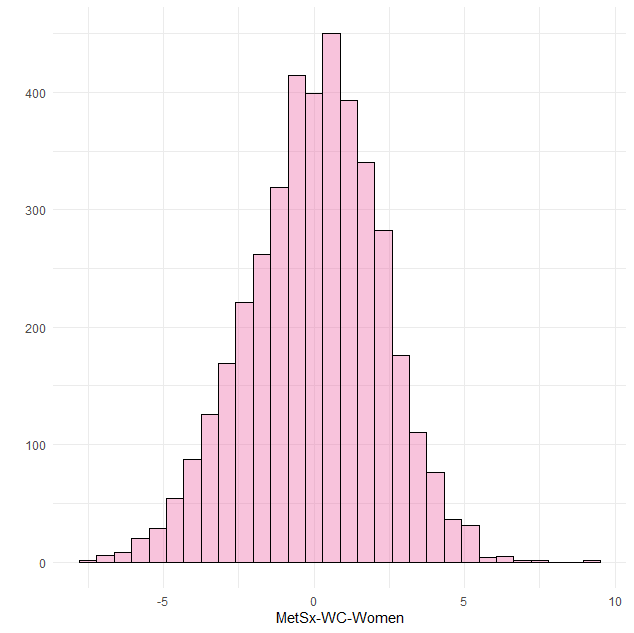 | | | | | 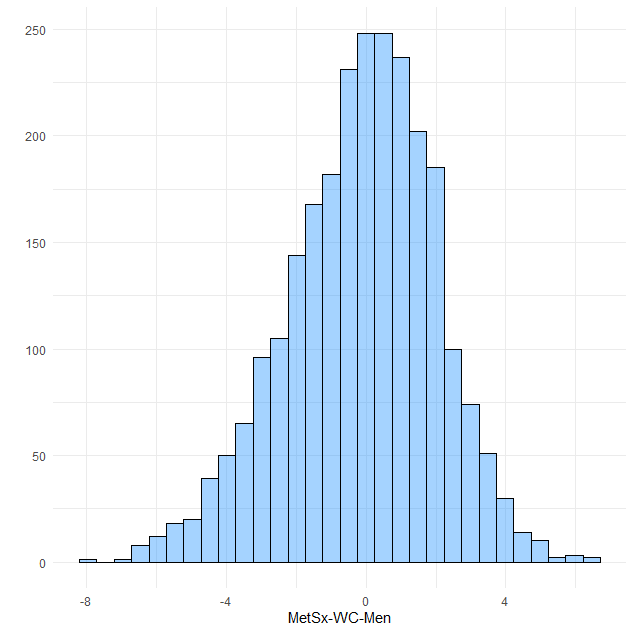 | | | | |
| --- | --- | --- | --- | --- | --- | --- | --- | --- | --- |
| S3 A. Histogram of women’s MetSx-WC scores by ENSANUT 2018. | | | | | S3 B. Histogram of men’s MetSx-WC scores by ENSANUT 2018. | | | | |
| Mean | SD | Q1 | Q2 | Q3 | Mean | SD | Q1 | Q2 | Q3 |
| 0.02 | 2.19 | -1.38 | 0.15 | 1.53 | -0.14 | 2.12 | -1.50 | 0.02 | 1.31 |

Supplemental Figure 3. Histograms of MetSx-WC score by sex.

| 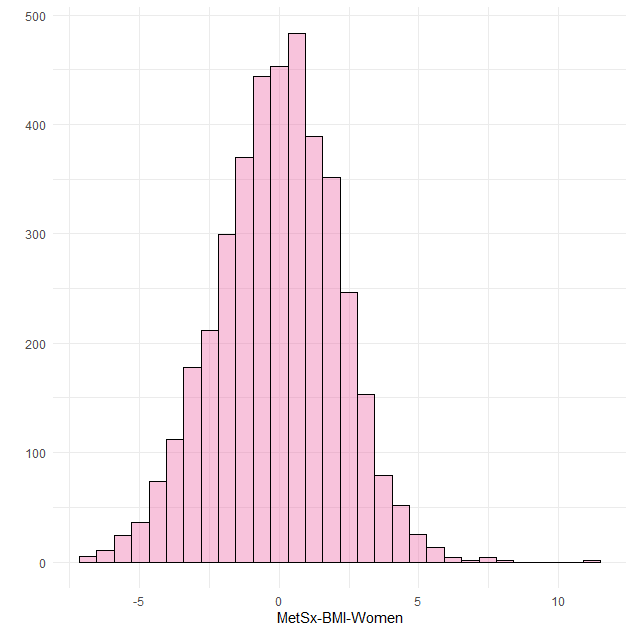 | | | | | 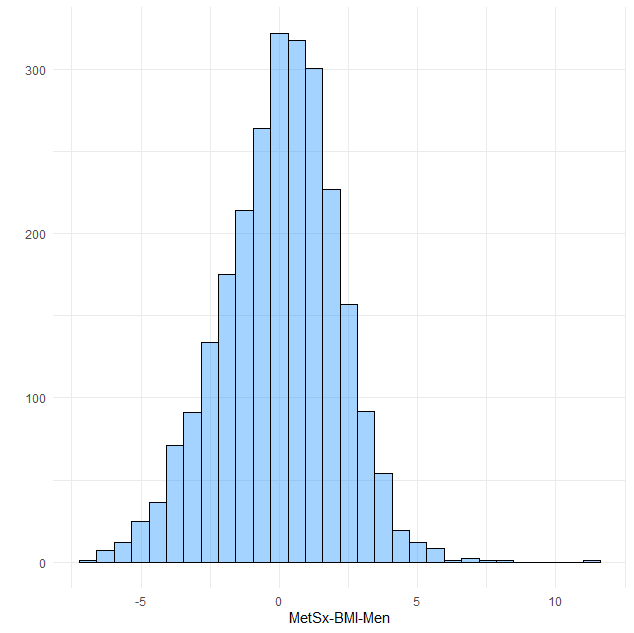 | | | | |
| --- | --- | --- | --- | --- | --- | --- | --- | --- | --- |
| S4 A. Histogram of women’s MetSx-BMI scores by ENSANUT 2018. | | | | | S4 B. Histogram of men’s MetSx-BMI scores by ENSANUT 2018. | | | | |
| Mean | SD | Q1 | Q2 | Q3 | Mean | SD | Q1 | Q2 | Q3 |
| -0.05 | 2.16 | -1.42 | 0.08 | 1.45 | 0.02 | 2.10 | -1.31 | 0.14 | 1.45 |

Supplemental Figure 4. Histograms of MetSx- BMI score by sex.

**References**

[S1] Geer EB, Shen W. Gender Differences in Insulin Resistance, Body Composition, and Energy Balance. Gend Med 2009;6:60. https://doi.org/10.1016/J.GENM.2009.02.002.

[S2] Barquera S, Rivera JA. Obesity in Mexico: rapid epidemiological transition and food industry interference in health policies. Lancet Diabetes Endocrinol 2020;8:746–7. https://doi.org/10.1016/S2213-8587(20)30269-2.

[S3] Gayoso-Diz P, Otero-Gonzalez A, Rodriguez-Alvarez MX, Gude F, Cadarso-Suarez C, García F, et al. Insulin resistance index (HOMA-IR) levels in a general adult population: Curves percentile by gender and age. The EPIRCE study. Diabetes Res Clin Pract 2011;94:146–55. https://doi.org/10.1016/j.diabres.2011.07.015.

[S4] Wolin KY, Colangelo LA, Chiu BC-H, Gapstur SM. Obesity and Immigration Among Latina Women. J Immigr Minor Health 2009;11:428. https://doi.org/10.1007/S10903-007-9115-1.
